# A randomized, double-blind, parallel control study to evaluate the biosimilarity of QL1209 with Perjeta^®^ in healthy male subjects

**DOI:** 10.3389/fphar.2022.953641

**Published:** 2022-08-23

**Authors:** Yuanyuan Sun, Heng Yang, Xiaoyan Yang, Shuang Yang, Can Guo, Honghui Chen, Chang Cui, Yuxia Xiang, Guoping Yang, Jie Huang

**Affiliations:** ^1^ Center of Clinical Pharmacology, The Third Xiangya Hospital, Central South University, Changsha, China; ^2^ Department of Neurology, The Third Xiangya Hospital of Central South University, Changsha, China; ^3^ Research Center of Drug Clinical Evaluation of Central South University, Changsha, China; ^4^ Department of Pharmacy, The Third Xiangya Hospital, Central South University, Changsha, China; ^5^ Xiangya School of Pharmaceutical Sciences, Central South University, Changsha, China; ^6^ National-Local Joint Engineering Laboratory of Drug Clinical Evaluation Technology, Changsha, China

**Keywords:** QL1209, pertuzumab, biosimilar, pharmacokinetics, safety, immunogenicity

## Abstract

**Purpose:** This is the first study to compare the pharmacokinetics, safety and, immunogenicity of QL1209, a biosimilar of Perjeta^®^.

**Methods:** This study was a randomized, double-blind, parallel-controlled clinical trial evaluating the biosimilarity between QL1209 (specification: 420 mg:14 ml, single use *via*, manufacturer: Qilu Pharmaceutical Co., Ltd., batch number: 201808001KJL) and Perjeta^®^ (specification: 420 mg: 14 ml, single use *via*, manufacturer: Roche Pharma AG, batch number: H0309H02). The trial period was 99 days (blood samples for PK were collected 99 days after infusion). Serum concentrations were determined using a validated assay. PK parameters were calculated using a non-compartmental model and analyzed statistically. Anti-drug antibody (ADA)-positive samples were further tested for the presence of neutralization antibody detection (NAb).

**Results:** A total of 137 healthy subjects were administrated. The subjects were randomized 1:1 to receive QL1209 or Perjeta^®^ 420 mg intravenously. The geometric mean ratio (GMRs) for QL1209 versus Perjeta^®^ are 104.14%, 104.09%, and 110.59% for C_max_, AUC_0-t_, and AUC_0-∞_, respectively, and their 90% confidence interval (CIs) all fell within the predefined bioequivalence margin 80.00–125%. The incidence of drug-related adverse events was 95.6% and 95.5% in the QL1209 and Perjeta^®^ groups, respectively, also comparable between the two groups.

**Conclusion:** The results of this comparative clinical pharmacology study demonstrated the PK similarity of QL1209 (420 mg: 14 ml) and Perjeta^®^ (420 mg: 14 ml) and there was no significant difference in safety and immunogenicity between QL1209 and Perjeta^®^ manufactured by Roche Pharma AG.

## 1 Introduction

Breast cancer, one of the most common malignant tumors in women, leads to thousands of deaths each year (Cesca et al., 2020). Breast cancer is a heterogeneous disease composed of several biological subtypes; for instance, luminal A breast cancer, luminal B breast cancer, human epidermal growth factor receptor 2 (HER2)-enriched breast cancer, triple-negative breast cancer, and normal-like breast cancer (Feng et al., 2018). Each has distinct behaviors, treatment strategies, clinical results and prognoses. HER2-positive breast cancer is an aggressive subtype accounting for approximately 15–20% of breast cancer cases (Loibl and Gianni, 2017; Agarwal et al., 2021). HER2, also known as Erythroblastosis homolog B2 (ErbB-2), is a member of human EGFR family with four domains, I, II, III, and IV, in the N-terminal extracellular region (Nitta et al., 2016; Mahmoudi et al., 2021). HER2 overexpression can lead to downstream signaling pathway overactivation, associate with increasing tumor cell proliferation and enhancing tumor invasiveness and angiogenesis, result in the pathogenesis and progression of solid tumors (Bartlett et al., 2001; Maennling et al., 2019; Henriques et al., 2021).

The current standard of care is dual blockade with trastuzumab + pertuzumab as the first-line treatment, followed by ado-trastuzumab emtansine (T-DM1) as second-line (Cesca et al., 2020). Trastuzumab and pertuzumab are recombinant humanized anti-HER2 monoclonal antibodies bind to extracellular domain IV (ECD IV) and domain II (ECD II) of HER2, respectively, interfere the formation of heterodimerization of other ErbB members, block downstream signaling pathways and inhibit cancer cell proliferation. Pertuzumab (840 mg loading dose followed by 420 mg every 3 weeks, single intravenous injection infusion over 30–60 min) was initially approved by the FDA in 2012, now it is used in combination with trastuzumab and docetaxel to treat patients with HER2-positive metastatic breast cancer (MBC) and a neoadjuvant treatment for patients with HER2-positive, locally advanced, inflammatory, or early-stage breast cancer (either greater than 2 cm in diameter or node-positive) at high risk of recurrence (Amiri-Kordestani et al., 2014; Howie et al., 2019). Pertuzumab was approved for use in China in 2018, and many clinical trials are ongoing. Developing a biosimilar medicine could help to improve treatment availability and decrease medical expenses. As such, this is of great significance for meeting Chinese patients’ health needs.

No biosimilar drugs to pertuzumab have been approved until now, though many similar drugs have been developed. QL1209, developed by Qilu Pharmaceutical Co., Ltd., is a biosimilar drug to Perjeta^®^, which has shown a high degree of similarity to Perjeta^®^ in both quality and properties presented in preclinical studies. Therefore, we conducted a randomized, double-blind, parallel-controlled comparative clinical pharmacology study to compare the pharmacokinetics, safety, tolerability, and immunogenicity of QL1209 (420 mg: 14 ml) injection and Perjeta^®^ (420 mg: 14 ml) injection in healthy male subjects. The result of this study indicated the PK similarity of QL1209 (420 mg: 14 ml) and Perjeta^®^ (420 mg: 14 ml), and the groups did not differ significantly in safety and immunogenicity.

## 2 Materials and methods

### 2.1 Participants and eligibility

From 2018 to 2020, 137 healthy male subjects were enrolled, while 134 in 137 male subjects completed the dosing. All subjects met the inclusion criteria, including age between 18 and 55, weight no less than 50 kg, and body mass index (BMI) within 19–26. Before the study, all subjects provided written informed consent. This trial was approved by the Institutional Review Board of the Third Xiangya Hospital, Central South University (CTR20182330), and abided by ethics principles of the Declaration of Helsinki and Good Clinical Practice (GCP) guidelines.

### 2.2 Study design

The study was designed as a randomized, double-blind, parallel-controlled study to compare the pharmacokinetics, safety, tolerability, and immunogenicity of QL1209 injection (specification: 420 mg:14 ml, single use *via*, manufacturer: Qilu Pharmaceutical Co., Ltd., batch number: 201808001KJL) and Perjeta^®^ injection (specification: 420 mg: 14 ml, single use *via*, manufacturer: Roche Pharma AG, batch number: H0309H02) in healthy male subjects. The primary objective was to compare the pharmacokinetics (PK) between the QL1209 and Perjeta^®^. The secondary objective was to evaluate the safety and immunogenicity of QL1209. The subjects were divided into two groups in a 1:1 ratio randomly with Interactive Web Response System and Statistical Analysis System. Then the subjects of the QL1209 and Perjeta^®^ groups received their assigned medication intravenously (single intravenous injection infusion 420 mg over 60 min).

#### 2.2.1 Pharmacokinetic evaluations

Blood samples for PK analysis were collected at 0 (before drug administration), 0.5 (during drug administration), 0 (after drug administration), 0.5, 1.5, 3, 4, 8, 12, 24 (day 1), 48 (day 2), 96 (day 4), 168 (day 7), 336 (day 14), 504 (day 21), 672 (day 28), 1,008 (day 42), 1,344 (day 56), 1,512 (day 63), 1,680 (day 70), 2,016 (day 84), and 2,352 (day 98) hours. Blood samples were left standing for 30–60 min at room temperature and centrifuged at 1,500 g for 15 min at 4°C. Then, the serum was stored at −80°C for further analysis. Enzyme-linked immunosorbent assays (ELISA) were used to analyze QL1209 and Perjeta^®^ concentrations in the serum.

#### 2.2.2 Safety evaluations

We assessed treatment safety by the following metrics (Cesca et al., 2020): Any adverse events or serious adverse events including those self-reported and directly observed (Feng et al., 2018); Any abnormal changes in vital signs and physical examination (Loibl and Gianni, 2017); Abnormal laboratory examination, electrocardiogram, transabdominal ultrasound, or ultrasonic cardiogram during the trial (Agarwal et al., 2021); Immunogenicity assessment according to the number and percentage of subjects producing anti-drug antibodies (ADA) and neutralization antibodies (NAbs) detection. We monitored adverse events (AE) through systemic organ classification (SOC) and preferred term (PT) classifications according to MedDRA 23.1.

#### 2.2.3 Immunogenicity evaluations

We collected immunogenicity analysis blood samples on day 1 (before drug administration), then days 15, 29, 43, 71, and 99 after drug administration to measure ADA and NAb incidence. Only the ADA-positive samples would undergo further testing for the presence of NAbs. We assessed ADA′ and NAb′ presence by electrochemiluminescence immunoassay (ECLIA). The number and percentage of subjects who produced ADAs and NAbs were summarized by group. In the subjects who were still positive for ADAs/NAbs at the last visit of the trial, immunogenicity samples were collected for further follow-up of antibody outcomes at the third and sixth months.

### 2.3 Statistical design

According to prior PK study results of Perjeta^®^ (Committee for Medicinal Products for Human Use (CHMP), 2012), effective sample size was 138 (Assuming intragroup coefficient of variation: 39%, intergroup area under curve (AUC) ratio: 1.05, power (1-beta): 85%; significance (alpha): 5%, two-sided, equivalence margin: 80.00–125%). The pharmacokinetic analysis was calculated using Phoenix^®^ WinNonlin^®^ 8.0 (Certara, Princeton, NJ, USA), and the statistical analysis used SAS Enterprise Guide 9.4. The pharmacokinetic parameters, including maximum observed serum concentration (C_max_), AUC from zero to the time of the last quantifiable concentration (AUC_0–t_), AUC from zero extrapolated to infinity (AUC_0-∞_), clearance (CL), apparent volume of distribution (V_d_), and terminal half-life (T_1/2_) were calculated based on individual serum concentration-time data and actual sampling time for each subject. Subgroup analyses by ADA and NAb were also conducted. We used an analysis of variance (ANOVA) model after the pharmacokinetic parameters’ logarithmic transformation. Then, PK biosimilarity between QL1209 and Perjeta^®^ was evaluated with and without bodyweight as a stratification factor, with a 90% confidence interval (CI) as the tests’ power. If 90% CI of AUC_0-t_ geometric mean ratio (GMR) between two drugs was within 80.00–125%, QL1209 injection could be considered equivalent to Perjeta^®^ in PK.

We descriptively analyzed safety and immunogenicity. Safety evaluation included (Cesca et al., 2020) the frequency of changes of laboratory examination indices and the list of abnormal and clinically significant laboratory examination indices after treatment, and (Feng et al., 2018) the frequency, number, incidence, and severity of adverse events and adverse reactions in each group. Immunogenicity was evaluated by the number and percentage of ADA-positive and NAb-positive subjects according to the groups and visits, and the occurrence time and duration of ADA-positive were descriptively analyzed.

## 3 Results

### 3.1 Demographic characteristics

**FIGURE 1:**
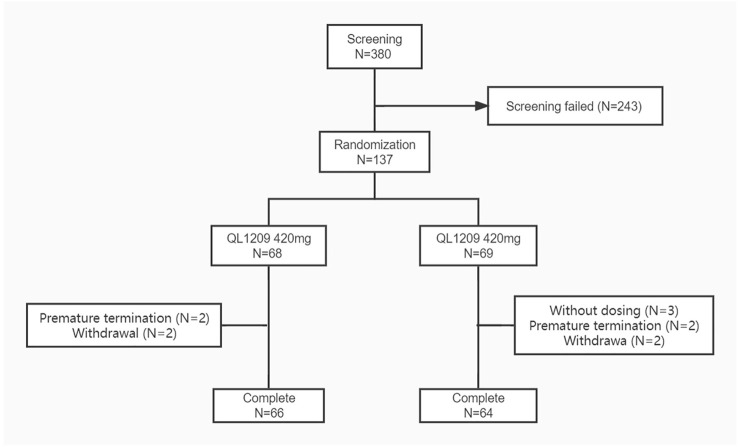
Subject flow chart. N: The number of subjects. Screening failed mean subjects who did not meet the inclusion criteria or exclusion criteria.

137 healthy Chinese male subjects were enrolled, while 134 received the assigned study drugs and completed the trial (Figure 1). The demographic and baseline characteristics of all subjects are presented in Table 1. There were no significant differences between the demographic and baseline parameters among the QL1209 and Perjeta^®^ groups. Among the 137 subjects in this research, 125 subjects finished the detection of PK concentration which included 22 blood collection points for each subject as planned. Four subjects finished the detection of PK concentration which included 21 blood collection points. So, it is considered to exist 129 valid PK concentration data which accounted for 93.5% of the effective sample size. It is evaluated statistically that the effective samples have been obtained presently.

**TABLE 1:** Demographic and baseline characteristics of all the subjects.

	QL1209 420 mg (N = 68)	Perjeta^®^ 420 mg (N = 66)
Age (Years)		
Mean (±SD)	24.5 (4.85)	22.4 (3.77)
Range	18–36	18–35
Male (%)	68 (100%)	66 (100%)
Height (cm)		
Mean (±SD)	1.6904 (0.0593)	1.6911 (0.0651)
Range	1.575–1.820	1.545–1.850
Weight (kg)		
Mean (±SD)	63.71 (6.189)	62.55 (6.636)
Range	51.2–80.2	50.0–80.2
Body mass index (kg/m2)
Mean (±SD)	22.31 (1.952)	21.85 (1.713)
Range	19.2–25.9	19.2–25.7
Ethnicity		
Han	60 (88.2%)	61 (92.4%)
Other	8 (11.8%)	5 (7.6%)

### 3.2 Pharmacokinetic evaluations

**FIGURE 2:**
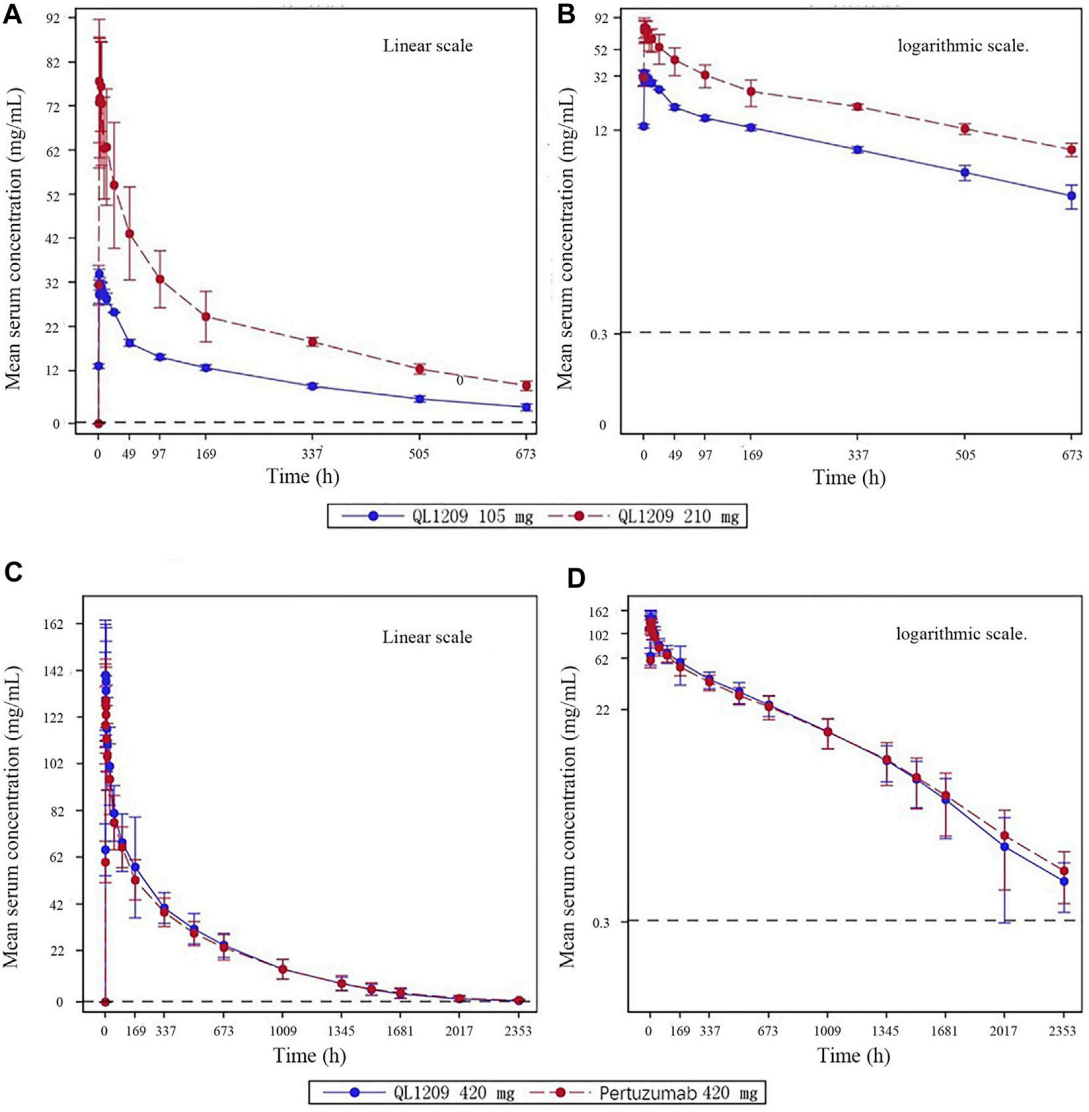
Mean blood concentration time curve. **(A)** Mean plasma concentration (±SD) time curve after intravenous drip of QL1209 105 mg or 210 mg. **(B)** Logarthimic transformation of the mean plasma concentration (±SD) time curve after intravenous dripping of QL1209 105 mg or 210 mg. **(C)** Mean plasma concentration (±SD) time curve after intravenous dripping of QL1209 420 mg or Perjeta^®^ 420 mg. **(D)** Logarthimic transformation of the mean plasma concentration (±SD) time curve after intravenous dripping of QL1209 420 mg or 420 mg.

126 in 137 subjects were included in the pharmacokinetic analysis set. The PK parameters of Perjeta^®^ and QL1209 are shown in Table 2, demonstrating that QL1209 had a slightly lower T_1/2_ and V_d_ than Perjeta^®^ and a slightly higher in AUC_0-t_, AUC_0-∞_, C_max_, and T_max_. According to the statistical comparison of pharmacokinetic parameters given in Table 3, the GMRs for QL1209 versus Perjeta^®^ were 104.14%, 104.09%, and 110.59% for C_max_, AUC_0-t_, and AUC_0-∞_, and 104.32%, 104.27%, and 110.63% for C_max_, AUC_0-t_, and AUC_0-∞_, respectively, with bodyweight as a stratification factor. The GMRs for QL1209 versus Perjeta^®^ and their 90% CIs all fell within the predefined bioequivalence margin of 80.00–125%. The mean serum concentration-time profiles of Perjeta^®^ and QL1209 are given in Figure 2, which exhibited a similar trend. Finally, the drugs’ mean serum concentrations were close at each point in time. Above all, QL1209 is similar to Perjeta^®^ in PK.

**TABLE 2:** Summary of pharmacokinetic parameters for QL1209 and Perjeta^®^.

	QL1209 420 mg (N = 68) n (CV%)	Perjeta^®^ 420 mg (N = 66) n (CV%)	
Parameter (unit)			
AUC_0-t_ (h*ng/mL)	44,990 (19.8%)	43,093 (18.49%)	
AUC_0-∞_ (h*ng/mL)	45,296 (19.52%)	43,427 (18.45%)	
C_max_ (ng/ml)	147.63 (15.2%)	133.32 (13.11%)	
T_max_ (h)	5.38 (386.93%)	2.61 (42.85%)	
T_1/2_ (h)	175.89 (31.08%)	187.84 (24.7%)	
CL (ml/h)	9.63 (19.57%)	9.99 (18.21%)	
V_d_ (ml)	2440.9 (39.53%)	2677.4 (26.36%)	

**TABLE 3:** Statistical comparison of pharmacokinetic parameters.

Parameters (units)	Geometric means		Geometric means ratio % (90% CI)	Power (%)	T1		T2	
	QL1209	Perjeta^®^			Statistic	P	Statistic	P
Without stratification								
AUC_0-t_ (h*mg/mL)	44,139	42,385	104.14 (98.44–110.16)	99.99	7.77	<0.0001	5.38	<0.0001
AUC_0-∞_ (h*mg/mL)	44,463	42,717	104.09 (98.44–110.06)	>99.99	7.82	<0.0001	5.44	<0.0001
C_max_ (mg/ml)	146.03	132.16	110.49 (105.96–115.22)	99.93	12.78	<0.0001	4.88	<0.0001
Stratification by bodyweight									
AUC_0-t_ (h*mg/mL)	45,445	43,561	104.32 (99.12, 109.80)	>99.99					
AUC_0-∞_ (h*mg/mL)	45,766	43,890	104.27 (99.12, 109.70)	>99.99					
C_max_ (mg/ml)	149.09	134.76	110.63 (106.46, 114.97)	99.98					

T1, T2 is the results of one-sided *t* test. T1: Geometric means ratio of QL1209 420 mg and Perjeta^®^ ≤ 80%; T2: Geometric means ratio of QL1209 420 mg and Perjeta^®^ ≥ 80%.

### 3.3 Safety evaluations

A total of 573 treatment‐emergent adverse effects (TEAEs) related to QL1209 and Perjeta^®^ were reported from 128 (95.5%) subjects, summarized in Table 4. Most TEAEs were grade 1 or 2. Grade 3 or higher TEAEs [2 (2.9%) vs. 2 (3.0%)], SAEs [1 (1.5%) vs. 1 (1.5%)], and adverse events of concern (80.9% vs. 83.3%) were comparable in the QL1209 and Perjeta^®^ groups. There were no dose reductions, discontinuations, withdrawals, or deaths due to TEAEs.

**TABLE 4:** Summary of treatment‐emergent adverse events and drug-related TEAEs by category in part-2.

Systemic organ classification preferred term	QL1209 420 mg (N = 68)		Perjeta^®^ 420 mg (N = 66)		Total (N = 134)	
	n (%)	(nAE)	n (%)	(nAE)	n (%)	(nAE)
TEAEs	67 (98.5%)	532	65 (98.5%)	583	132 (98.5%)	1,115
TEAEs related to study drugs	65 (95.6%)	270	63 (95.5%)	303	128 (95.5%)	573
TEAEs≥ 3 grade	2 (2.9%)	2	2 (3.0%)	3	4 (3.0%)	5
Dose reduction due to TEAEs	0	0	0	0	0	0
Suspension due to TEAEs	1 (1.5%)	1	0	0	1 (0.7%)	1
Discontinuations due to TEAEs	0	0	0	0	0	0
Withdrawal due to TEAEs	0	0	0	0	0	0
Death due to TEAEs	0	0	0	0	0	0
SAE	1 (1.5%)	1	1 (1.5%)	1	2 (1.5%)	2
SAE related to study drugs	1 (1.5%)	1	0	0	1 (0.7%)	1
Adverse events needed attention	55 (80.9%)	93	55 (83.3%)	99	110 (82.1%)	192
Gastrointestinal	51 (75.0%)	118	51 (77.3%)	125	102 (76.1%)	243
Diarrhea	38 (55.9%)	45	37 (56.1%)	50	75 (56.0%)	95
Oral ulcers	29 (42.6%)	44	27 (40.9%)	41	56 (41.8%)	85
Bellyache	7 (10.3%)	7	11 (16.7%)	11	18 (13.4%)	18
Hematochezia	1 (1.5%)	1	4 (6.1%)	4	5 (3.7%)	5
Skin and subcutaneous tissue	47 (69.1%)	68	49 (74.2%)	62	96 (71.6%)	130
Rash	41 (60.3%)	47	46 (69.7%)	49	87 (64.9%)	96
Skin stripped	5 (7.4%)	5	5 (7.6%)	5	10 (7.5%)	10
Infectious	19 (27.9%)	25	31 (47.0%)	43	50 (37.3%)	68
Upper respiratory infection	13 (19.1%)	15	27 (40.9%)	36	40 (29.9%)	51
Paronychia	3 (4.4%)	3	5 (7.6%)	5	8 (6.0%)	8
Nervous system	19 (27.9%)	20	24 (36.4%)	27	43 (32.1%)	47
Dysgeusia	16 (23.5%)	16	23 (34.8%)	25	39 (29.1%)	41
Respiratory system, chest and mediastinum	15 (22.1%)	20	13 (19.7%)	18	28 (20.9%)	38
Epistaxis	6 (8.8%)	7	8 (12.1%)	9	14 (10.4%)	16
Oropharyngeal pain	4 (5.9%)	4	1 (1.5%)	2	5 (3.7%)	6
Ocular	6 (8.8%)	6	3 (4.5%)	3	9 (6.7%)	9
Blurred vision	4 (5.9%)	4	2 (3.0%)	2	6 (4.5%)	6
Psychotropic	2 (2.9%)	2	6 (9.1%)	6	8 (6.0%)	8
Insomnia	2 (2.9%)	2	6 (9.1%)	6	8 (6.0%)	8

TEAEs, treatment‐emergent adverse events; nAE, number of adverse events (the unit is time); SAEs, serious adverse events.

Number (%) of subjects more than 5% in each group is included. Percentage calculations are based on the number of subjects in each group.

Adverse events that cannot be determined or missed are also considered as investigational drug related adverse events. The bold values mean “Systemic organ classification”.

The most frequent incidences of QL1209 and Perjeta^®^ in drug-related TEAEs are as follows: rash (60.3% vs. 69.7%, respectively), diarrhea (55.9% vs. 56.1%), oral ulcers (42.6% vs. 40.9%), upper respiratory tract infections (19.1% vs. 40.9%), dysgeusia (23.5% vs. 34.8%), bellyache (10.3% vs. 16.7%) and epistaxis (8.8% vs. 12.1%). The incidence of rashes, upper respiratory tract infections, and dysgeusia was lower in the QL1209 group than the Perjeta^®^ group, while other drug-related adverse events occurred almost equally between the two groups. In summary, QL1209 is similar to Perjeta^®^ in safety and tolerability.

### 3.4 Immunogenicity evaluations

**FIGURE 3:**
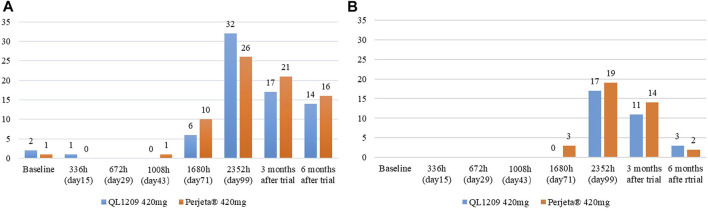
**(A)** The number of ADA-Positive subjects for QL1209 and Perjeta^®^ at baseline, 336 h (day 15), 672 h (day 29), 1008 h (day 43), 1680 h (day 71), 2352 h (day 99), 3 months after trail and 6 months after trail. **(B)** The number of Nab-Positive subjects for QL1209 and Perjeta^®^ at baseline, 336 h (day 15), 672 h (day 29), 1008 h (day 43), 1680 h (day 71), 2352 h (day 99), 3 months after trail and 6 months after trail.

In this study, three subjects were positive for ADAs at baseline, two with antibodies against QL1209 and one with antibodies against Perjeta^®^, details showed in Figure 3. Of the ADA-negative subjects at baseline, 45.5% (30/66) and 38.5% (25/65) of the subjects were positive for ADA in the QL1209 and Perjeta^®^ groups, respectively, after treatment administration. Without considering baseline subjects with ADA, there were slightly more ADA-positive subjects after baseline in the QL1209 group than in the Perjeta^®^ group (47.1% vs. 39.4%). However, this difference decreased gradually at 3 and 6 months after the trial’s end (25.0% vs. 31.8%, 20.6% vs. 24.2%). The median ADA-positive time was 99 days, both in the QL1209 and Perjeta^®^ groups, and the geometric regularity of antibody titers of ADA-positive subjects in the QL1209 group was similar to that of the Perjeta^®^ group at each visit point. These results indicate that QL1209 had a similar ADA profile to Perjeta^®^ in this study.

All subjects were negative for NAb at baseline. NAb-negative subjects mainly occurred on the 99th day and the last follow-up. NAb-positive subjects were slightly less common in the QL1209 group than the Perjeta^®^ group. Of the ADA-negative subjects at baseline, 28.8% (19/66) and 30.8% (20/65) of subjects were positive for NAbs in the QL1209 group and the Perjeta^®^ group, respectively, after baseline. After taking the ADA and NAb into control, the pharmacokinetic parameters were still similar, suggesting that ADA do not affect the PK values (Table 5).

**TABLE 5:** Summary of pharmacokinetic parameters for QL1209 and Perjeta^®^ analyses by ADA and NAb subgroup.

	QL1209 420 mg (N = 68)		Perjeta^®^ 420 mg (N = 66)	
Pharmacokinetics analyzed by ADA subgroup				
	Positive n (CV%)	Negative n (CV%)	Positive n (CV%)	Negative n (CV%)
AUC_0-t_ (h*mg/mL)	45,220 (20.17%)	44,800 (19.76%)	44,836 (18.88%)	41,916 (17.9%)
AUC_0-∞_ (h*mg/mL)	45,565 (19.69%)	45,074 (19.65%)	45,192 (18.89%)	42,235 (17.8%)
C_max_ (mg/ml)	150.55 (15.42%)	145.21 (15.02%)	137.46 (11.93%)	130.53 (13.67%)
T_max_ (h)	8.39 (368.72%)	2.89 (43.88%)	2.76 (45.54%)	2.5 (40.59%)
T_1/2_ (h)	170.77 (37.8%)	180.13 (25.21%)	180.32 (29.74%)	192.92 (21.15%)
CL (ml/h)	9.56 (19.21%)	9.69 (20.11%)	9.61 (18.52%)	10.25 (17.8%)
V_d_ (ml)	2386.2 (51.68%)	2486.3 (27.51%)	2440 (26.36%)	2837.8 (24.98%)
Pharmacokinetics analyzed by NAb subgroup				
	Positive n (CV%)	Negative n (CV%)	Positive n (CV%)	Negative n (CV%)
AUC_0-t_ (h*mg/mL)	47,457 (20.25%)	40,970 (16.08%)	43,750 (19.8%)	49,180 (13.51%)
AUC_0-∞_ (h*mg/mL)	47,715 (20.06%)	41,480 (15.05%)	44,100 (19.78%)	49,560 (13.69%)
C_max_ (mg/ml)	156.45 (15.83%)	139.35 (11.07%)	137.41 (13.14%)	137.68 (5850.79%)
T_max_ (h)	11.41 (334.65%)	2.64 (55.28%)	2.97 (44.04%)	1.93 (27.38%)
T_1/2_ (h)	163.25 (17.63%)	185.06 (56.58%)	174.13 (25.68%)	205.08 (40.21%)
CL (ml/h)	9.15 (20.85%)	10.33 (14.34%)	9.86 (18.48%)	8.61 (15.03%)
V_d_ (ml)	2152.6 (26.19%)	2830 (68.53%)	2426 (25.02%)	2496 (34.18%)
TEAEs and SAE analyzed by ADA subgroup				
	Positive	Negative	Positive	Negative
TEAEs	31 (96.9%)	36 (100%)	26 (100%)	39 (97.5%)
TEAEs related to study drugs	30 (93.8%)	35 (97.2%)	26 (100%)	37 (92.5%)
TEAEs ≥ 3 grade	1 (3.1%)	1 (2.8%)	0	2 (5.0%)
Suspension due to TEAEs	1 (3.1%)	0	0	0
SAE	1 (3.1%)	0	0	1 (2.5%)
SAE related to study drugs	1 (3.1%)	0	0	0
Adverse events needed attention	28 (87.5%)	27 (75.0%)	21 (80.8%)	34 (85.0%)

## 4 Discussion

When developing biosimilar therapeutic monoclonal antibodies, clinical pharmacokinetics (PK), efficacy, safety, and immunogenicity comparisons are essential (Ishii-Watabe and Kuwabara, 2019). According to the results of this study, the PKs of QL1209 (420 mg: 14 ml) and Perjeta^®^ (420 mg: 14 ml) were equivalent, and there was no significant difference in safety and immunogenicity between QL1209 and Perjeta^®^. Because this was the first-in-human study of QL1209, we conducted an exploratory study to evaluate the safety and tolerability in six subjects and evaluated PK characteristics preliminarily. PK profiles of 105 and 210 mg doses in our exploratory study showed similar trends (Figure 2). No dose reductions, discontinuations, withdrawals, serious adverse effects (SAEs), or deaths due to TEAE occurred, with all TEAEs grade 1–2 AEs. Notably, one subject tested positive for ADAs on day 29 in the QL1209 105 mg group. Further NAb testing on day 124 returns negative. In summary, these findings provided evidence supporting further evaluation of QL1209.

Previous publications reported that the maintenance dose of Perjeta^®^ was 420 mg administered every 3 weeks (Swain et al., 2020; Gianni et al., 2012; von Minckwitz et al., 2017), Perjeta^®^’s pharmacokinetics demonstrated a linear relationship within the dose range of 2–25 mg/kg (Yamamoto et al., 2009), and its half-life was approximately 20 days (11.1–22.3 days). Hence, we selected 420 mg as the dosage in our formal trial, and day 99 was our last blood collection (about five half-lives). As the mean ADA-positive diagnosis was generally found on day 15–20 after drug administration, the first blood sampling time was day 15, and the last was day 99, giving ample time to fully evaluate immunogenicity changes. We utilized single-dose intravenous administration to efficiently demonstrate differences in PK and PK parameters between the two groups. Since previous PK data showed no significant difference in efficacy between genders (Garg et al., 2014), healthy male volunteers were selected in our study.

Our results demonstrated that QL1209 has a similar PK profile to Perjeta^®^. The GMRs and 90% CIs for QL1209 versus Perjeta^®^ fell within the predefined bioequivalence margin of 80.00–125%. All tests’ powers were more than 99%. The analysis stratified by the ADA status and bodyweight further demonstrated PK similarity between QL1209 and Perjeta^®^.

Most of the TEAEs that occurred were grade 1 or 2. Rates of grade 3 TEAEs or higher, adverse events of concern, and SAE were similar between the QL1209 and Perjeta^®^ groups, supporting our conclusion of comparable safety profiles in both drugs. Moreover, we found that six patients experienced blurred vision (a symptom of optic nerve injury) in our study, which have not been reported previously in FDA’s approval of Pejeta. In our study, four patients experienced blurred vision in QL1209 group and two in Perjeta^®^. Japanese drug directions for Perjeta^®^ have also included this adverse drug reaction (ADR), but the incidence (<2%) was too low to include in the European, US, and Chinese drug directions. Previous studies of anti-HER2 monoclonal antibodies have reported blurred vision as an adverse event (Papageorgiou et al., 2021; Sterenczak et al., 2021). Some studies have attributed this symptomatology to corneal lesion development, mostly reversible (Kreps et al., 2018; Deklerck et al., 2019; Sharma et al., 2021). Corneal nerves are part of the peripheral nervous system, and peripheral neuropathy was observed as a common adverse reaction (>30%) in the FDA’s approval of Perjeta^®^ (Blumenthal et al., 2013; Howie et al., 2019). Due to high vascularity, rapid proliferation, and abundant receptor distribution for various signaling molecules, the eye is particularly susceptible to the effects of targeted agents (Ho et al., 2013). Thus, clinicians should pay more attention to possible ophthalmological complications during anti-HER2 monoclonal antibody therapy (Ma et al., 2022). The median ADA-positivity time in the QL1209 group was approximately equivalent to that of the Perjeta^®^ group, and the ADA-positive subjects’ antibody titers’ geometric regularity in the QL1209 group was similar to the Perjeta^®^ group at each visit point, indicating that QL1209 had a similar ADA profile to Perjeta^®^ in this study.

## 5 Conclusion

The results of this comparative clinical pharmacology study demonstrate the PK similarity of QL1209 (420 mg: 14 ml) and Perjeta^®^ (420 mg: 14 ml), and there was no significant difference in safety and immunogenicity between QL1209 and Perjeta^®^.

## Data Availability

The data that support the findings of this study are available from the corresponding author, GY, upon reasonable request.
